# Economic Evaluation of Mailed Home-Based Human Papillomavirus Self-sampling Kits for Cervical Cancer Screening

**DOI:** 10.1001/jamanetworkopen.2023.4052

**Published:** 2023-03-22

**Authors:** Richard T. Meenan, Catherine Troja, Diana S. M. Buist, Jasmin A. Tiro, John Lin, Melissa L. Anderson, Hongyuan Gao, Beverly B. Green, Rachel L. Winer

**Affiliations:** 1 Center for Health Research, Kaiser Permanente Northwest, Portland, Oregon; 2 Department of Epidemiology, University of Washington School of Public Health, Seattle; 3 Kaiser Permanente Washington Health Research Institute, Seattle; 4 Department of Clinical Sciences, The University of Texas Southwestern Medical Center, Dallas; 5 Washington Permanente Medical Group, Seattle; 6 Department of Health Systems Science, Kaiser Permanente Bernard J. Tyson School of Medicine, Pasadena, California

## Abstract

**Importance:**

Human papillomavirus (HPV) self-sampling addresses barriers to cervical cancer screening, and mailed self-sampling kits have been reported to increase screening uptake. International research suggests mailed kits are cost-effective in certain settings. However, the cost-effectiveness of mailing HPV self-sampling kits for increasing screening uptake has not been evaluated in the US.

**Objective:**

To conduct an economic evaluation of a mailed HPV self-sampling intervention among underscreened women enrolled in an integrated US health care system.

**Design, Setting, and Participants:**

This economic evaluation involved a cost-effectiveness analysis of results from a randomized clinical trial of 19 851 women aged 30 to 64 years enrolled in a health plan from Kaiser Permanente Washington (KPWA), a US-based integrated health care system. Women were identified through electronic medical records, and eligible participants were enrolled in a health plan for at least 3 years and 5 months, had a primary care clinician, had not received a Papanicolaou test for at least 3 years and 5 months, and had not received a hysterectomy. Enrollment occurred from February 25, 2014, to August 29, 2016, with follow-up through February 25, 2018. The current economic evaluation was conducted between August 2, 2021, and July 30, 2022. Intervention delivery costs were calculated from both the KPWA and Medicare perspectives and were based on either wellness visit or Papanicolaou test–only visit costs.

**Intervention:**

Participants in the control group received usual care, which comprised patient reminders and ad hoc outreach for screening. Participants in the intervention group received usual care plus a mailed HPV self-sampling kit.

**Main Outcome and Measures:**

The primary economic outcome was the incremental cost-effectiveness ratio (ICER) for increased screening uptake, defined as the incremental difference in cost (intervention group minus control group) divided by the difference in the number of participants completing screening (intervention group minus control group) within 6 months of randomization.

**Results:**

Among 19 851 women (mean [SD] age, 50.1 [9.5] years; 76.7% White), 9960 were randomized to the intervention group, and 9891 were randomized to the control group. Baseline ICERs ranged from $85.84 (95% CI, $85.68-$85.99) using KPWA wellness visits as the cost basis to $146.29 (95% CI, $146.20-$146.38) using Medicare Papanicolaou test–only visits as the cost source. Subgroups of participants aged 50 to 64 years and participants most recently overdue for screening achieved cost-effectiveness at lower levels of willingness to pay for an additional completed screening than other subgroups.

**Conclusions and Relevance:**

In this economic evaluation, mailing HPV self-sampling kits to women overdue for cervical cancer screening was cost-effective for increased screening uptake relative to usual care. These results support mailing HPV kits as an efficient outreach strategy for increasing screening rates among eligible women in US health care systems.

## Key Points

**Question:**

What is the cost-effectiveness of mailing human papillomavirus self-sampling kits relative to usual care for increasing cervical cancer screening rates among underscreened female members of an integrated US health care system?

**Findings:**

In this economic evaluation involving 19 851 female participants from a randomized clinical trial, incremental cost-effectiveness ratios for increased screening uptake ranged from $86 to $146 per additional completed screening.

**Meaning:**

These findings suggest that within US-based private integrated health care systems, mailing human papillomavirus self-sampling kits to individuals who are overdue for cervical cancer screening is cost-effective and may be an efficient outreach strategy to increase screening rates among eligible women.

## Teaser

This economic evaluation uses data from a randomized clinical trial to assess the cost-effectiveness of a mailed human papillomavirus self-sampling kit for increasing screening uptake among underscreened women enrolled in an integrated private US health care system.

## Introduction

Cervical screening, primarily through Papanicolaou (ie, cytologically based) testing, has been associated with substantial global reductions in cervical cancer incidence and mortality.^1^ Most cervical cancers are preventable by addressing precancers caused by high-risk human papillomavirus (HPV) and thus occur predominantly in individuals who have never or rarely received screening.^2^ Recent advances allow for HPV-only (primary HPV) screening, which is more sensitive than a Papanicolaou test alone for detecting precancerous cervical lesions (eg, grade 2 or higher cervical intraepithelial neoplasia [CIN2+]).^3,4,5^ Home-based HPV-only screening is feasible because, unlike Papanicolaou tests, both clinicians and individuals can collect vaginal HPV samples. Vaginal samples are accurate for detecting CIN2+ and use the same HPV assay as cervical HPV samples.^6^ Home-based screening reduces the need for office visits and addresses well-documented barriers to regular screening, including Papanicolaou-related discomfort or embarrassment and difficulty scheduling or attending medical appointments.^7,8^ Globally, a systematic review^9^ of randomized clinical trials has suggested direct mailing of home-based HPV self-collection kits was associated with increases in screening uptake among underscreened women, although most trials in the review were conducted in settings with organized national screening programs.

To generate data on the effectiveness of mailed HPV kits for cervical cancer screening within a US-based health care system, the Home-Based Options to Make Cervical Cancer Screening Easy (HOME) randomized clinical trial was conducted within Kaiser Permanente Washington (KPWA), an integrated private health care system in Washington State.^10^ The HOME trial compared a mailed HPV self-sampling intervention with KPWA usual care outreach for in-clinic screening. Screening uptake increased among underscreened women receiving mailed HPV kits compared with those receiving usual care, supporting the feasibility of mailing HPV kits to women overdue for screening as an effective outreach strategy.^11^

However, successfully implementing an HPV self-sampling program requires understanding how home-based HPV testing affects health system resources and the cost-effectiveness of home-based kits in particular settings. Most studies in a 2020 systematic review^12^ found HPV self-sampling programs to be cost-effective. Typically, the level of increase in screening attendance was associated with increased cost-effectiveness, with lower material and testing costs, longer duration of underscreening among self-sampling kit users, and higher sensitivity to detect cervical precancer, which are also important factors.^12^ However, to date, most HPV self-sampling cost-effectiveness research has been conducted alongside randomized clinical trials in European settings with organized national screening programs.^12,13,14,15,16,17,18^ In such settings, larger than expected increases in colposcopy referrals were observed, increasing overall screening costs. Because less coordinated US health care systems have adopted primary HPV screening after 2018 and 2020 updates to national cervical cancer screening guidelines^19,20^ and begun to consider implementation strategies including self-sampling programs, the cost-effectiveness of home-based HPV screening programs for underscreened individuals within such systems should be evaluated. In this economic evaluation, we performed a cost-effectiveness analysis of increased screening uptake using results from the HOME trial.

## Methods

### Setting and Participants

Study design, recruitment details, and results from the underlying clinical trial have been published previously.^10,11^ In the HOME clinical trial, 19 851 eligible individuals were randomized 1:1 to the control or intervention group between February 25, 2014, and August 29, 2016, with follow-up through February 25, 2018. For the current economic evaluation, participant-level economic data were collected between June 2, 2019, and March 31, 2021, and data analysis was conducted between August 2, 2021, and July 30, 2022. The institutional review boards (IRBs) of KPWA and the University of Washington approved all study procedures for the clinical trial and the current economic evaluation. The IRBs provided a waiver of informed consent for this economic evaluation because it was deemed to be of minimal risk to participants. This study followed the Consolidated Health Economic Evaluation Reporting Standards (CHEERS) reporting guideline for economic evaluations.^21^

In the HOME trial, potential participants were identified using electronic medical records (EMRs) and determined to be eligible if they were female, aged 30 to 64 years, had a KPWA primary care physician, were continuously enrolled for at least 3 years and 5 months (to allow individuals the opportunity to receive screening after annual reminders), had not had a hysterectomy, and had not had a Papanicolaou and/or high-risk HPV test within 3 years and 5 months. Time since the most recent Papanicolaou test for each participant, including evidence of no previous test, was derived from the EMR. Individuals who previously indicated they did not want to be contacted for research studies, were pregnant, or had an EMR-based *interpreter needed* flag (HPV kit materials were written in the English language) were excluded.

Given documented racial and ethnic disparities in cervical cancer screening in the US,^22^ data on race and ethnicity were also collected and analyzed to identify potential differences in intervention effectiveness across groups. Race and ethnicity at randomization were derived from self-reported EMR data; categories included American Indian or Alaska Native, Asian, Black or African American, Native Hawaiian or other Pacific Islander, White, other race and/or ethnicity (those selecting the *other* category were able to enter a free-text description; IRB approval did not allow reporting of individual-level data for all participants, so to maintain consistency across all participants in categorization of race and ethnicity, free-text entries were not reviewed or coded), and unknown race and/or ethnicity.^23^

### HOME Trial Intervention and Procedures

Participants in the HOME control group received usual care, which involved outreach to attend Papanicolaou screening, including a birthday letter reminder, clinician-targeted alerts signaling overdue screening that remained active until overridden or a Papanicolaou procedure was ordered, and communication from primary care teams. Participants in this group did not receive the mailed HPV kit and were not contacted by the study team.

Participants in the HOME intervention group received usual care plus a mailed HPV self-sampling kit with a prepaid return envelope addressed to the KPWA clinical laboratory. Mailings included an invitation letter, a research information sheet, and educational materials on self-collecting and returning a sample. The letter advised participants to receive routine Papanicolaou testing regardless of whether they chose to complete the HPV self-sampling kit. When the HOME trial was initiated, cervical cancer screening guidelines did not yet include primary HPV screening. Therefore, the KPWA Institutional Review Board and delivery system required that participants be advised to attend in-clinic screening regardless of whether they chose to complete the self-sampling kit. More recent guidelines recommend HPV testing for primary screening.^19^ The letter clarified that participation was voluntary and provided a telephone number for recipients to call with questions or to opt out of having their EMR data used for research. If the kit was not returned within 3 weeks, study personnel made up to 3 reminder calls, consistent with KPWA outreach protocols. Samples collected in clinical care and from kit self-collection were evaluated using the same test (Cobas 4800 HPV Amplification/Detection Kit; Roche Diagnostics). Results were documented in the EMR, and primary care teams managed communication of screening results and follow-up care. Participants with negative or unsatisfactory results were advised to attend in-clinic screening (Papanicolaou-only testing or Papanicolaou and HPV cotesting). Participants with positive results for high-risk HPV types other than HPV type 16 (HPV-16) or HPV type 18 (HPV-18) were advised to receive in-clinic cotesting. Participants with positive results for HPV-16 or HPV-18 were recommended for immediate colposcopy per published guidelines.^24^ Clinical and system-level collaborators developed standardized protocols to educate primary care teams about recommended follow-up (protocols were published previously^10^).

### Outcomes

Using EMR data, 2 primary outcomes were assessed in the HOME trial: histologically diagnosed CIN2+ and treated CIN2+. Diagnosed CIN2+ was captured within 6 months after an abnormal screening result that occurred within 6 months after randomization, and treated CIN2+ was captured within 6 months after a diagnosis of CIN2+. Each participant was followed up to 18 months after randomization.

The current economic evaluation focused on the secondary HOME trial outcome of screening uptake, which was captured by the receipt of cervical cancer screening within 6 months after randomization and defined as receiving (1) in-clinic screening, (2) a positive HPV-16 or HPV-18 result on a self-sampled HPV test, (3) a negative result on a self-sampled HPV test, or (4) a positive result on a self-sampled HPV test for types other than HPV-16 or HPV-18 or an unsatisfactory result on subsequent in-clinic screening. This definition of screening uptake allowed either modality (self-sampling kit or in-clinic screening) to count as an outcome. Focusing on an intermediate outcome, such as screening uptake, was deemed more salient to US-based health care systems and payers. The primary outcome of this economic evaluation was the incremental cost-effectiveness ratio (ICER) for screening uptake.

### Cost Measurement

Intervention costs were defined as the value of resources used to implement the mailed HPV kit program during the HOME trial and were measured from the health care system perspective. A microcost analysis was used to assess intervention resources, and resources were classified as labor or nonlabor. Intervention cost data came from expense reports, the KPWA cost management database, and external sources (eg, the 2021 Medicare Physician Fee Schedule^25^). Papanicolaou test–related visit costs were estimated across 2 dimensions: (1) a KPWA-based or Medicare-based perspective and (2) a wellness visit (ie, a primary care appointment to manage a comprehensive prevention plan) or a Papanicolaou test–only visit basis. These dimensions captured cost differences between private and public funders as well as differences in the clinical context of Papanicolaou procedures. We used *Current Procedural Technology* codes to develop cost estimates for event-based laboratory processing and screening visits. Although costs of primary care visits and colposcopies were higher when services were provided through KPWA vs reimbursed by Medicare, processing costs of KPWA testing were not universally higher than those from the Medicare Physician Fee Schedule (eg, HPV test processing with a normal or positive result for HPV-16 or HPV-18 was $35.09 through Medicare vs $26.64 through KPWA).

We also focused on eligible women who were overdue for routine cervical cancer screening; therefore, once screened, a participant was removed from the screening pool. Although abnormal screening results would induce subsequent follow-up costs (eg, a mild abnormal case in 1 year would require a repeat cotesting in 12 months), we excluded these costs. We focused on the cost-effectiveness of the HOME intervention in increasing the rate of completed screening uptake. In addition, the low (12.0%) proportion of abnormal cases requiring surveillance in the sample (1.5% of those who received usual care, 5.2% of those who received a mailing and a direct in-clinic Papanicolaou test, and 4.2% of those who received a mailing and a self-sampling HPV test) implied that follow-up costs would not materially change the results given the sample size. Costs were not discounted because of the 18-month study period but were adjusted to 2021 values using the medical care Consumer Price Index for urban consumers.^26^

### Statistical Analysis

The primary outcome, ICER for screening uptake, was calculated as the difference in cost (intervention condition minus control condition) divided by the difference in the number of participants completing screening (intervention group minus control group) within 6 months of randomization. Inherent uncertainty in the ICERs was assessed by 95% CIs using Fieller’s theorem.^27,28^ Cost-effectiveness acceptability curves assessing the probability of the intervention condition being cost-effective relative to the control condition at various willingness to pay (WTP) values for an additional completed screening were also generated for EMR-derived subgroups (based on age [30-39 years, 40-49 years, or 50-64 years] and time since last Papanicolaou test [>3.4 to <5.0 years, ≥5.0 years to <10.0 years, ≥10.0 years, or no previous test]) using nonparametric methods over 1000 bootstrap replications. In these analyses, the outcome was additional completed screening uptake. All analyses used Stata software, version 16 (StataCorp LLC).^29^

## Results

### HOME Trial Results

Among 19 851 female participants (mean [SD] age, 50.1 [9.5] years; 1773 Asian [9.7%], 869 Black or African American [4.7%], and 14 129 White [76.7%]) included in the intention-to-treat analysis, 9960 were randomized to the intervention group, and 9891 were randomized to the control group. Baseline characteristics between groups were without significant differences (eTable in Supplement 1).^11^ Screening uptake was higher in the intervention group vs the control group (2618 participants [26.3%] vs 1719 participants [17.4%]; relative risk, 1.51; 95% CI, 1.43-1.60). Within the intervention group, 1440 participants received a Papanicolaou test directly in the clinic. Mailed HPV kits were returned by 1206 participants; 1178 of these participants met criteria for completed screening uptake. Twelve participants in the intervention group had positive results for CIN2+ compared with 8 participants in the control group (relative risk, 1.49; 95% CI, 0.61-3.64), and 12 participants in the intervention group received treatment for CIN2+ vs 7 participants in the control group (relative risk, 1.70; 95% CI, 0.67-4.32).

### Economic Results

Unit costs of HOME trial intervention components are shown in Table 1, 12-month intervention costs by study group are shown in Table 2, and baseline ICERs distinguished by cost basis (KPWA vs Medicare) and visit type (wellness vs Papanicolaou testing only) are shown in Table 3. The ICERs for the total sample ranged from $85.84 (95% CI, $85.68-$85.99) per additional completed screening using KPWA-based wellness visit costs to $146.29 (95% CI, $146.20-$146.38) for using Medicare-based Papanicolaou test–only visit costs (Table 3). Narrow 95% CIs surrounding the baseline ICERs were due to the large sample size. These ICERs, which represented the mean cost of an additional completed screening incurred by the HOME trial intervention vs usual care, were comparable with, if not lower than, many individual procedure unit costs (eg, baseline ICER for KPWA wellness visit cost basis vs assigned cost of a KPWA wellness visit: $85.84 vs $357.97 for ages 18-39 years or $365.24 for ages 40-64 years; baseline ICER for Medicare wellness visit cost basis vs assigned cost of a Medicare wellness visit: $125.28 vs $162.65 for ages 18-39 years or $171.65 for ages 40-64 years) (Table 1).

**Table 1.** Intervention Unit Costs

Intervention unit	Primary care visit cost, $^a^	
	Medicare	KPWA
**Age group, y**		
18-39		
Wellness visit	162.65	357.97
Papanicolaou test–only visit	84.09	118.85
40-64		
Wellness visit	171.65	365.24
Papanicolaou test–only visit	84.09	118.85
**Mailing**		
First	14.73	14.73
Second	14.73	14.73
**Processing**		
Cotesting		
Any abnormal result on Papanicolaou or HPV test	77.84	101.44
Any normal or unsatisfactory result on Papanicolaou or HPV test	55.35	47.04
Normal or positive result for HPV-16 and/or HPV-18 on HPV test kit only^b^	35.09	26.64
HPV test kit result		
Other positive result for high-risk HPV (negative result for HPV-16 and HPV-18) and normal or unsatisfactory result on Papanicolaou test	55.35	47.04
Other positive result for high-risk HPV (negative result for HPV-16 and HPV-18) and abnormal result on Papanicolaou test	77.84	101.44
Any unsatisfactory result on HPV test plus cotest and any normal or unsatisfactory result on Papanicolaou test and second HPV test	90.44	73.68
Colposcopy (plus biopsy and ECC)	319.41	488.18

Abbreviations: ECC, endocervical curettage; HPV, human papillomavirus; KPWA, Kaiser Permanente Washington.

^a^ Including Papanicolaou testing.

^b^ HPV-16 and HPV-18 are high-risk types known to significantly increase the risk of cervical, vaginal, and vulvar cancer in women.

**Table 2.** Total 12-Month Intervention Costs by Study Group

Intervention	Cost, $	
	Intervention group (n = 9960)	Control group (n = 9891)
Primary care visit		
Medicare		
Wellness	257 838	292 195
Papanicolaou test only	127 733	144 551
KPWA		
Wellness	552 459	625 528
Papanicolaou test only	180 414	204 303
Mailing		
First	146 711	0
Second	2990	0
Processing		
Medicare	87 474	96 294
KPWA	76 978	83 636
Colposcopy		
Medicare	19 803	12 457
KPWA	30 267	19 039
Total cost		
Medicare		
Wellness visit	514 817	400 946
Papanicolaou test–only visit	384 711	253 301
KPWA		
Wellness visit	809 405	728 204
Papanicolaou test–only visit	437 360	306 978
Cost per participant (95% CI)		
Medicare		
Wellness visit	51.69 (49.87-53.54)	40.54 (38.68-42.39)
Papanicolaou test–only visit	38.63 (37.30-39.95)	25.61 (24.34-26.88)
KPWA		
Wellness visit	81.27 (78.04-84.56)	73.62 (70.30-76.95)
Papanicolaou test–only visit	43.91 (42.13-45.70)	31.04 (29.36-32.70)

Abbreviation: KPWA, Kaiser Permanente Washington.

**Table 3.** Incremental Cost-effectiveness Ratios

Variable	Baseline KPWA and Medicare				KPWA					
	Primary care visit type	Incremental screening uptake rate	Incremental cost, $	ICER, $ (95% CI) per outcome	Participants, No.		Screening uptake rate, %		ICER, $ (95% CI) per outcome	
					Intervention group^a^	Control group	Intervention group	Control group	Wellness visit	Papanicolaou test–only visit
**Cost basis**										
KPWA	Wellness	0.089	7.64	85.84 (85.68 to 85.99)	NA	NA	NA	NA	NA	NA
	Papanicolaou test only	0.089	12.88	144.72 (144.63 to 144.81)	NA	NA	NA	NA	NA	NA
Medicare	Wellness	0.089	11.15	125.28 (125.11 to 126.44)	NA	NA	NA	NA	NA	NA
	Papanicolaou test only	0.089	13.02	146.29 (146.20 to 146.38)	NA	NA	NA	NA	NA	NA
**Age group, y**										
30-39										
Time since last Papanicolaou test, y										
No test	NA	NA	NA	NA	687	657	17.8	9.4	223.36 (−877.10 to 400.94)	241.07 (166.55 to 1693.11)
>3.4 to <5.0	NA	NA	NA	NA	834	839	34.9	29.1	236.89 (232.25 to 243.35)	186.81 (182.17 to 191.34)
≥5.0 to <10.0	NA	NA	NA	NA	204	199	24.5	14.1	−23.14 (−35.98 to −11.04)	162.10 (157.80 to 166.15)
≥10.0	NA	NA	NA	NA	15	14	13.3	7.1	280.41 (264.21 to 294.88)	247.98 (237.94 to 258.05)
40-49										
Time since last Papanicolaou test, y										
No test	NA	NA	NA	NA	790	794	13.2	8.7	288.65 (254.09 to 323.26)	306.59 (274.98 to 353.01)
>3.4 to <5.0	NA	NA	NA	NA	1251	1249	38.5	27.2	191.90 (184.27 to 198.79)	280.34 (276.01 to 284.42)
≥5.0 to <10.0	NA	NA	NA	NA	456	426	20.6	15.3	129.24 (126.11 to 132.15)	127.92 (127.40 to 130.25)
≥10.0	NA	NA	NA	NA	77	90	10.4	5.6	34.37 (13.88 to 51.93)	271.11 (261.80 to 281.17)
50-64										
Time since last Papanicolaou test, y										
No test	NA	NA	NA	NA	1799	1815	14.2	5.3	238.91 (233.45 to 244.98)	225.87 (222.04 to 229.98)
>3.4 to <5.0	NA	NA	NA	NA	2324	2342	38.2	28.5	199.06 (198.14 to 200.27)	192.95 (192.15 to 192.98)
≥5.0 to <10.0	NA	NA	NA	NA	1023	1064	24.1	11.7	−113.87 (−117.14 to −111.09)	66.23 (65.45 to 67.38)
≥10.0	NA	NA	NA	NA	383	402	13.1	4.2	129.95 (126.95 to 131.75)	130.24 (128.87 to 131.06)

Abbreviations: ICER, incremental cost-effectiveness ratio; KPWA, Kaiser Permanente Washington; NA, not applicable.

^a^ In the intervention group, 117 participants did not have demographic data.

The ICERs with 95% CIs stratified by age group and by years since last Papanicolaou procedure (KPWA cost basis) are shown in Table 3. Subgroup ICERs exhibited no particular pattern, although ICERs for groups with longer time since last Papanicolaou testing were generally lower than groups with shorter time since last testing (eg, ≥10.0 years vs >3.4 to <5.0 years among women aged 50-64 years with a KPWA-based wellness visit: $130 [95% CI, $127-$132] vs $199 [95% CI, $198-$200]).

**Figure 1.**
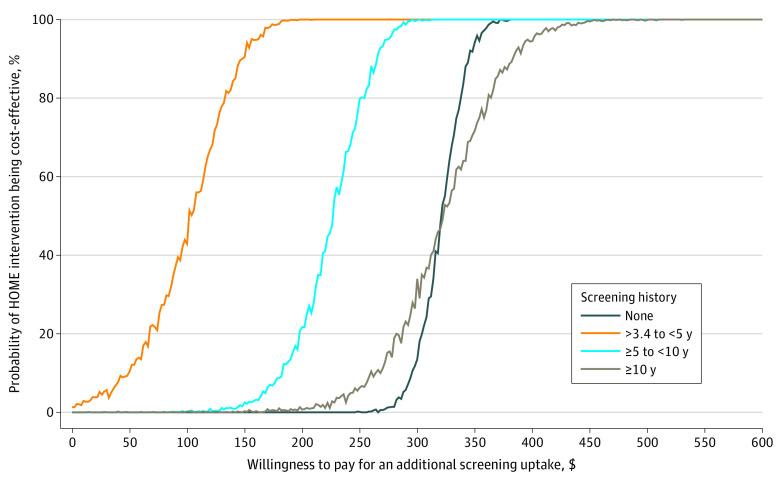
Kaiser Permanente Washington–Based Cost-effectiveness Acceptability Curves by Time Since Last Papanicolaou Test Based on wellness visit. HOME indicates Home-Based Options to Make Cervical Cancer Screening Easy randomized clinical trial.

**Figure 2.**
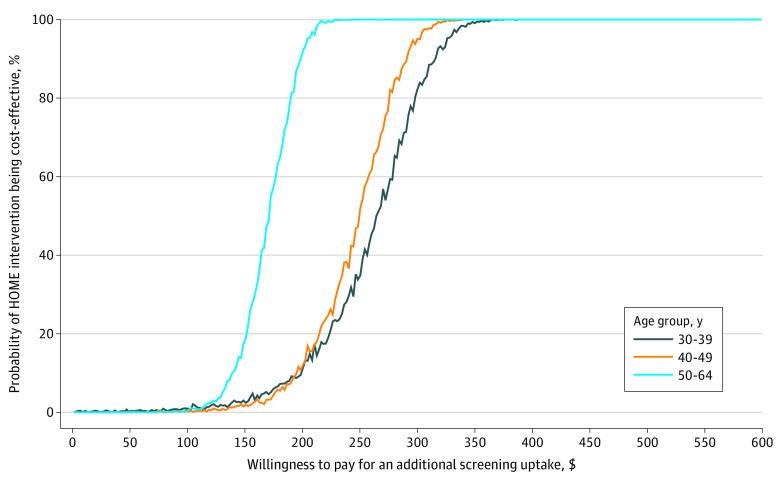
Kaiser Permanente Washington–Based Cost-effectiveness Acceptability Curves by Age Group Based on wellness visit. HOME indicates Home-Based Options to Make Cervical Cancer Screening Easy randomized clinical trial.

Cost-effectiveness acceptability curves for the intervention group compared with the control group stratified by screening history (ie, time since last Papanicolaou testing) are shown in Figure 1, and curves stratified by age group are shown in Figure 2. These figures show KPWA-based costs; Medicare-based costs are shown in eFigure 1 and eFigure 2 in Supplement 1. All figures represent results when wellness was used as the visit type. The intervention achieved cost-effectiveness at lower WTP levels among participants whose last Papanicolaou test was more than 3.4 years to less than 5.0 years before randomization (eg, 90% probability of cost-effectiveness at WTP of $148) than among participants in other subgroups (eg, ≥10 years since last Papanicolaou test: 90% probability of cost-effectiveness at WTP of $384) (Figure 1). At WTP values greater than $450, intervention cost-effectiveness was virtually certain (ie, approximately 100% probability) among all subgroups. Similarly, the intervention achieved cost-effectiveness at lower WTP values among participants aged 50 to 64 years (eg, 90% probability of cost-effectiveness at WTP of $198) than among other age groups (eg, aged 30-39 years: 90% probability of cost-effectiveness at WTP of $316) (Figure 2). At WTP values greater than $350, the intervention was virtually certain to be cost-effective (ie, 100% probability) across age groups.

## Discussion

In this economic evaluation of results from a randomized clinical trial conducted within a US-based integrated health care system, our mailed HPV self-sampling intervention generated additional completed cervical cancer screenings at an incremental cost from $85.84 to $146.29, depending on the cost basis used (KPWA or Medicare). The intervention achieved cost-effectiveness at lower system WTP levels among subgroups of women aged 50 to 64 years and those whose last Papanicolaou test was more than 3.4 years to less than 5.0 years before randomization than other subgroups.

Although our analysis incorporated clinic visit costs associated with Papanicolaou procedures, triage strategies are currently being studied that may incorporate testing positive self-samples with additional molecular markers to avoid a clinic visit.^30^ Economic analyses of such strategies may become increasingly relevant given the possibility that in-clinic triage by cytological assessment will become unnecessary for positive self-samples.

A previous HPV self-collection systematic review^12^ of 16 cost-effectiveness studies found that HPV self-sampling programs can be cost-effective for increasing cervical cancer screening uptake among underscreened individuals based on findings in 14 of the 16 studies. Our current analysis within a US-based integrated health care system with mailed HPV outreach for enrolled individuals supports this conclusion. Although subgroups of older participants and those with more recent screening history had lower values of WTP for an additional completed screening, cost-effectiveness was achieved at similar WTP levels across all subgroups.

Our study focused on assessing the cost-effectiveness of the HOME trial intervention in increasing the proportion of eligible women receiving appropriate cervical cancer screening within a US-based private health care system. Eligible studies included in the previous systematic review^12^ differed in target population, delivery methods, model type, and approach to triage of positive results. As in the HOME trial, source studies for 6 analyses were various randomized clinical trials of a self-sampling strategy targeting underscreened adult women.^13,14,15,16,17,18^ An important distinction of our analysis relative to those studies^13,14,15,16,17,18^ was our focus on the intermediate outcome of screening uptake rather than final outcomes of CIN2+ diagnosis and treatment and/or related quality-adjusted life-years saved, a measure not commonly used by US-based health care systems. Of the 6 trial-based studies^13,14,15,16,17,18^ in the systematic review,^12^ 4 specified uptake rate as the screening definition.^13,14,16,17^ Although their economic outcomes varied (cost per quality-adjusted life-year, cost per CIN2+ case treated, cost per CIN2+ case detected, or cost per extra woman screened), the self-screening uptake rate across these studies ranged from 17.0%^17^ to 31.3%.^13^ The comparable rate of 26.3% achieved among participants receiving the HPV self-sampling intervention in the HOME trial fits within this range.

Our results regarding the cost-effectiveness of an HPV self-sampling intervention were also consistent with those obtained in similar studies conducted in other settings and studies^31,32,33^ published after the systematic review.^12^ In a trial-based cost-effectiveness analysis,^31^ a self-sampling intervention was found to be cost-effective compared with a midwife-collected cytological sampling intervention among Swedish women with varied screening histories. In a model-based study in Malaysia,^32^ a digital registry of screening history was associated with enhanced cost-effectiveness of a combined HPV self-sampling and vaccination program compared with combined self-sampling and vaccination in the absence of such a registry. In addition, an economic model^33^ developed in connection with a group randomized clinical trial found that giving African American women the choice between HPV self-sampling screening and standard of care screening was cost-effective relative to standard of care screening alone.

Our analysis benefitted from the randomized clinical trial design and a large sample size that allowed us to evaluate the cost-effectiveness of the HOME intervention and to conduct relevant subgroup analyses (ie, age and screening history). Including KPWA and Medicare cost bases allowed comparability between representative US-based private and public payers. We also included the underlying clinic visit cost associated with a Papanicolaou procedure. Although not strictly part of the mailed HPV test intervention, the visit was an important resource associated with any Papanicolaou procedure performed within the trial.

The HOME study was conducted before the beginning of the COVID-19 pandemic in March 2020. It is well established that cancer screening rates internationally, including cervical cancer screening rates, decreased substantially during the pandemic.^34,35,36,37^ Societal consequences of the pandemic (eg, local stay-at-home restrictions) have highlighted the need for effective screening alternatives, especially for patients unwilling or unable to attend in-person appointments. The HOME trial results support using mailed HPV self-sampling kits as a means of overcoming screening barriers among underscreened women. Furthermore, our economic analysis adds to the evidence base supporting the cost-effectiveness of such mail-based programs.

### Limitations

This study has several limitations. It was conducted in 1 US region within a mixed-model health care system, which both insures and provides care within and outside of Kaiser Permanente. All study participants had some form of health insurance coverage and a primary care physician, which differs from the proportions of 88% with insurance coverage and 75% with a primary care physician among US adults.^38,39^ In addition, KPWA uses centralized systems for reminding members of the need for preventive care and provides multiple outreach strategies to encourage individuals who are overdue for screening to come in for services; this approach also differs from those used in health care systems in many other US settings. Therefore, our screening rates and follow-up after positive test results may differ from rates found in other settings. Although our analysis generates important US-based cost-effectiveness data, it does not address the lack of studies in low- and middle-income settings or predominantly rural settings.^12^ As noted in the previous systematic review^12^ of cervical cancer screening cost-effectiveness studies, economic analyses of mailed HPV self-screening in such settings remain important given the growing evidence of self-screening acceptability within communities of immigrants, women living in rural areas, and women with limited access to care.^40,41,42^

In addition, the HOME trial did not collect data on HPV vaccination history, limiting our ability to inform the association of cost-effectiveness with HPV vaccine uptake. However, the lack of such data was reasonable given that participants were women aged 30 to 64 years, most of whom would have been ineligible for HPV vaccination before the age of first sexual intercourse. The HPV vaccines were first available in the US in 2006 and were only recommended through age of 26 years before 2019.^43^ Although our analysis establishes the cost-effectiveness of mailed HPV self-sampling kits, it does not address the issue of the affordability of this program for health care systems, especially in the US. In future work, we will conduct a budget impact analysis evaluating the overall cost of implementing a HOME-type program within a health care system.

## Conclusions

In this economic evaluation of a program of mailing HPV self-sampling kits to women who were overdue for cervical cancer screening, the program was cost-effective relative to usual care in terms of increasing screening uptake at a reasonable cost within a private integrated health care system. These results support mailing HPV kits as an efficient outreach strategy for increasing screening rates in US health care systems.

## Notes

Supplement 1. **eTable.** Baseline Characteristics of Intervention and Control Group Participants **eFigure 1.** Medicare-Based Cost-effectiveness Acceptability Curves by Screening History **eFigure 2.** Medicare-Based Cost-effectiveness Acceptability Curves by Age Group

Supplement 2.
Data Sharing Statement
