# Supplementary material for: Economic Evaluation of Mailed Home-Based Human Papillomavirus Self-sampling Kits for Cervical Cancer Screening
Source: JAMA Netw Open. 2023 Mar 22;6(3):e234052. doi: 10.1001/jamanetworkopen.2023.4052 (PMC10034577; doi:10.1001/jamanetworkopen.2023.4052)
Supplement: Supplement 1. — eTable. Baseline Characteristics of Intervention and Control Group Participants eFigure 1. Medicare-Based Cost-effectiveness Acceptability Curves by Screening History eFigure 2. Medicare-Based Cost-effectiveness Acceptability Curves by Age Group [file jamanetwopen-e234052-s001.pdf]

# Supplementary Online Content

Meenan RT, Troja C, Buist DSM, et al. Economic evaluation of mailed home-based human papillomavirus self-sampling kits for cervical cancer screening. *JAMA Netw Open*. 2023;6(3):e234052. doi:10.1001/jamanetworkopen.2023.4052

**eTable.** Baseline Characteristics of Intervention and Control Group Participants

**eFigure 1.** Medicare-Based Cost-effectiveness Acceptability Curves by Screening History

**eFigure 2.** Medicare-Based Cost-effectiveness Acceptability Curves by Age Group

This supplemental material has been provided by the authors to give readers additional information about their work.

© 2023 Meenan RT et al. *JAMA Network Open*.

**eTable.** Baseline Characteristics of Intervention and Control Group Participants

| Characteristics[a] | Intervention (n = 9843)[b] | Control (n = 9891) |
|---|---|---|
| Age at randomization, y | | |
| 30-34 | 808 (8.2) | 794 (8.0) |
| 35-39 | 932 (9.5) | 915 (9.3) |
| 40-44 | 1194 (12.1) | 1185 (12.0) |
| 45-49 | 1380 (14.0) | 1374 (13.9) |
| 50-54 | 1682 (17.1) | 1707 (17.3) |
| 55-59 | 1938 (19.7) | 1943 (19.6) |
| 60-64 | 1909 (19.4) | 1973 (19.9) |
| Race[c] | | |
| White | 7018 (76.4) | 7111 (77.1) |
| Asian | 893 (9.7) | 880 (9.5) |
| Black or African American | 438 (4.8) | 431 (4.7) |
| Native Hawaiian or other Pacific Islander | 151 (1.6) | 139 (1.5) |
| American Indian/Alaska Native | 147 (1.6) | 145 (1.6) |
| >1 Race | 285 (3.1) | 283 (3.1) |
| Other | 250 (2.7) | 235 (2.5) |
| Unknown | 661 (6.7) | 667 (6.7) |
| Ethnicity[c] | | |
| Non-Hispanic | 8710 (94.7) | 8761 (94.8) |
| Hispanic | 486 (5.3) | 480 (5.2) |
| Unknown | 647 (6.6) | 650 (6.6) |
| Length of health plan enrollment, y | | |
| 3.4 to <5 | 2230 (22.7) | 2240 (22.6) |
| 5 to <10 | 3115 (31.6) | 3045 (30.8) |
| ≥10 | 4498 (45.7) | 4606 (46.6) |
| Time since last Papanicolaou test (by length of enrollment), y | | |
| Enrolled 3.4 to <5 y | | |
| No. | 2230 | 2240 |
| No Papanicolaou test | 1526 (68.4) | 1530 (68.3) |
| >3.4 to <5 | 704 (31.6) | 710 (31.7) |
| Enrolled 5 to <10 y | | |
| No. | 3115 | 3045 |
| No Papanicolaou test | 1056 (33.9) | 1070 (35.1) |
| >3.4 to <5 | 1519 (48.8) | 1468 (48.2) |
| 5 to <10 | 540 (17.3) | 507 (16.7) |
| Enrolled ≥10 y | | |
| No. | 4498 | 4606 |
| No Papanicolaou test | 694 (15.4) | 666 (14.5) |
| >3.4 to <5 | 2186 (48.6) | 2252 (48.9) |
| 5 to <10 | 1143 (25.4) | 1182 (25.7) |
| ≥10 | 475 (10.6) | 506 (11.0) |

© 2023 Meenan RT et al. *JAMA Network Open*.

| | | |
|---|---|---|
| Women's US Census block, median household income, median (IQR), $ | 66 474 (50 343-85 000) | 65 950 (50 536-83 949) |
| Travel time from women's home to primary care clinic, min | | |
| <10 | 3254 (33.4) | 3236 (33.1) |
| 10 to <20 | 4086 (41.9) | 4048 (41.4) |
| 20 to <30 | 1407 (14.4) | 1415 (14.5) |
| ≥30 | 1004 (10.3) | 1072 (11.0) |
| Unknown | 92 (0.9) | 120 (1.2) |
| Body mass index[d] | | |
| <18.5 | 109 (1.3) | 98 (1.2) |
| 18.5-24.9 | 2238 (26.5) | 2248 (26.7) |
| 25-29.9 | 2168 (25.7) | 2220 (26.3) |
| 30-34.9 | 1549 (18.4) | 1603 (19.0) |
| 35-39.9 | 1119 (13.3) | 1080 (12.8) |
| ≥40 | 1248 (14.8) | 1184 (14.0) |
| Unknown | 1412 (14.3) | 1458 (14.7) |
| Tobacco use | | |
| Never | 5237 (61.2) | 5232 (61.3) |
| Current | 1276 (14.9) | 1290 (15.1) |
| Former | 2041 (23.9) | 2020 (23.6) |
| Unknown | 1289 (13.1) | 1349 (13.6) |
| Charlson Comorbidity Index score[e] | | |
| 0 | 7967 (80.9) | 8052 (81.4) |
| 1 | 1087 (11.0) | 1128 (11.4) |
| 2 | 432 (4.4) | 385 (3.9) |
| ≥3 | 357 (3.6) | 326 (3.3) |
| Randomization year | | |
| 2014 | 4207 (42.7) | 4254 (43.0) |
| 2015 | 3570 (36.3) | 3571 (36.1) |
| 2016 | 2066 (21.0) | 2066 (20.9) |
| Abbreviation: IQR, interquartile range. | | |

[a]Based on electronic medical record (EMR) data.

[b]Baseline characteristics are not available for 117 participants who opted out of EMR review.

[c]Race and ethnicity from EMR data per patient self-report at usual care patient registration via preset multi-select categorical options, with "other" allowing free text entry. The study variable was programmatically categorized into the displayed categories by coding any multiple selections as "more than one race". Manual coding of the "other" category was precluded because IRB approval did not allow individual-level data for those opting out of study participation.

[d]Calculated as weight in kilograms divided by height in meters squared.

[e]Charlson ME, Carrozzino D, Guidi J, Patierno C. Charlson Comorbidity Index: A critical review of clinimetric properties. Psychother Psychosom. 2022;91:8–35.

© 2023 Meenan RT et al. *JAMA Network Open*.

**eFigure 1.** Medicare-Based Cost-effectiveness Acceptability Curves by Screening History

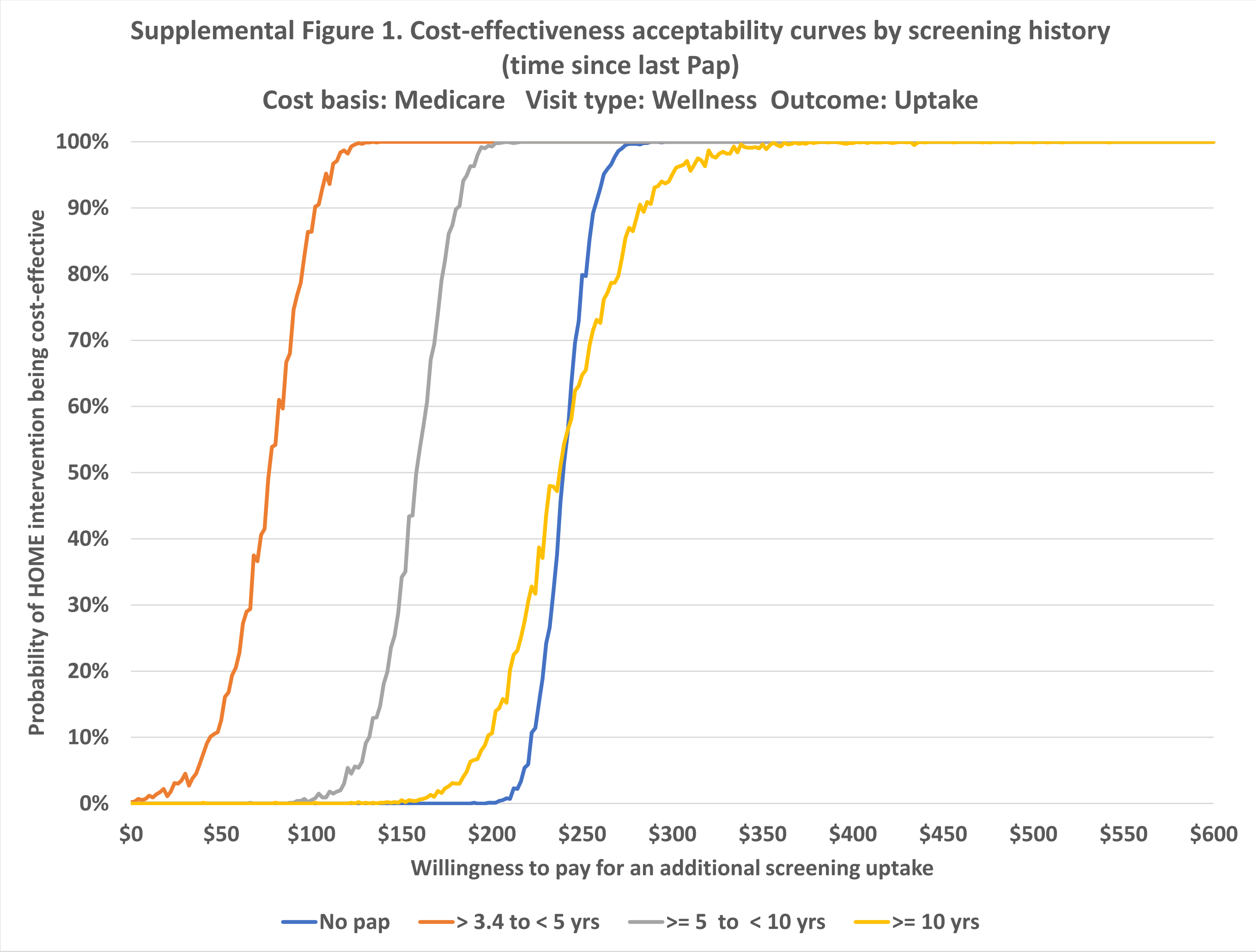


© 2023 Meenan RT et al. *JAMA Network Open*.

**eFigure 2.** Medicare-Based Cost-effectiveness Acceptability Curves by Age Group

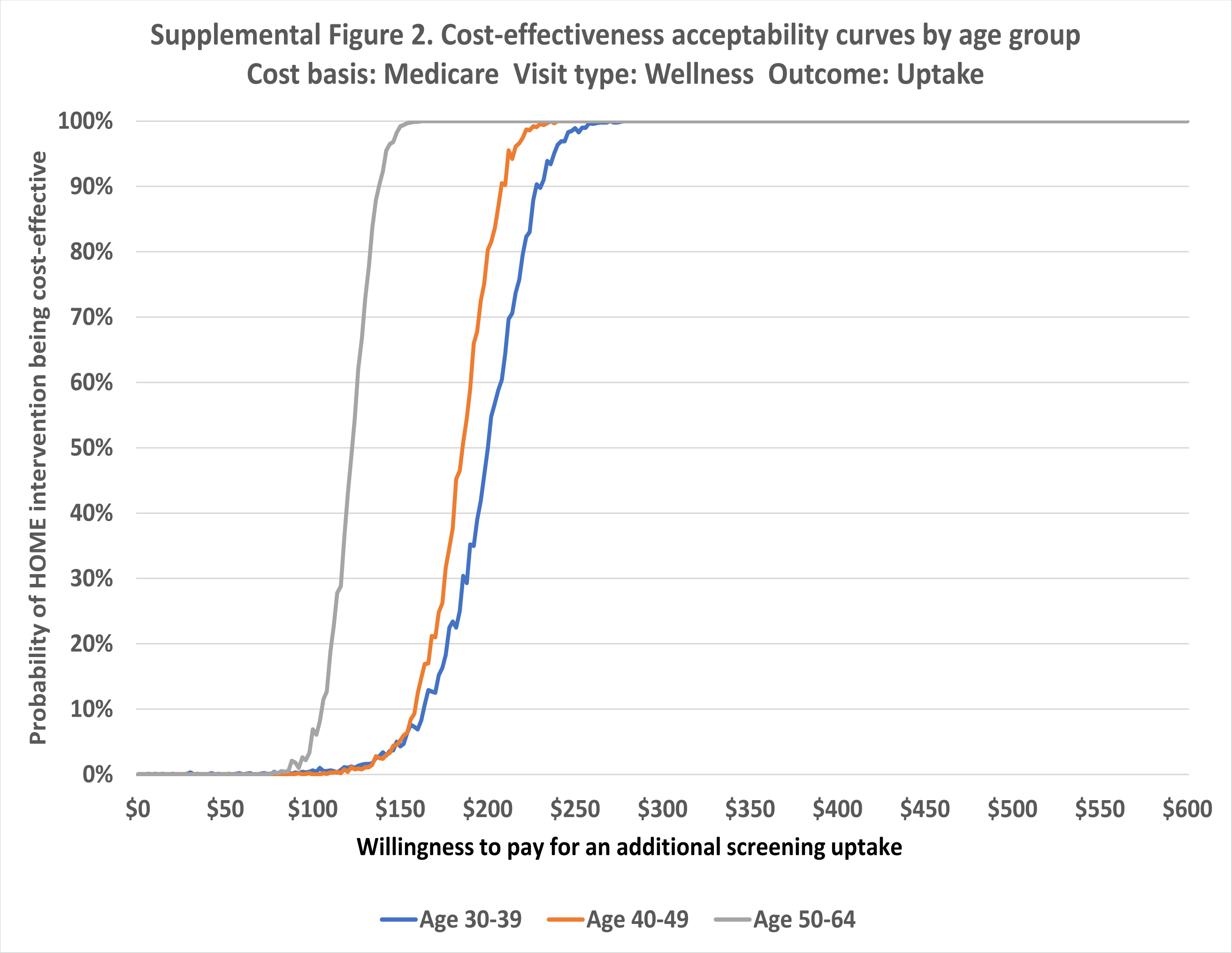


© 2023 Meenan RT et al. *JAMA Network Open*.
