# Supplementary material for: Economic Evaluation of Mailed Home-Based Human Papillomavirus Self-sampling Kits for Cervical Cancer Screening
Source: JAMA Netw Open. 2023 Mar 22;6(3):e234052. doi: 10.1001/jamanetworkopen.2023.4052 (PMC10034577; doi:10.1001/jamanetworkopen.2023.4052)
Supplement: Supplement 2. — Data Sharing Statement [file jamanetwopen-e234052-s002.pdf]

Data Sharing Statement

Meenan. Economic Evaluation of Mailed Home-Based Human Papillomavirus Self-Sampling Kits for Cervical Cancer Screening. *JAMA Netw Open*. Published March 22, 2023. doi:10.1001/jamanetworkopen.2023.4052

Data
Data available: Yes
Data types: Deidentified participant data, Data dictionary
How to access data: Data requests must be made to richard.meenan@kpchr.org.
When available: With publication

Supporting Documents
Document types: None

Additional Information
Who can access the data: Deidentified participant data and a data dictionary will be made available upon publication to researchers whose proposed use of the data has been approved.
Types of analyses: Data will be available for a clearly specified purpose only.
Mechanisms of data availability: Data will be made available after approval of a concept proposal aligned with current data approvals, and with a signed data access agreement.
Any additional restrictions: Data will be made available without investigator support to researchers with adequate resources to cover regulatory and data sharing costs.
